# Midkine inhibitors: application of a simple assay procedure to screening of inhibitory compounds

**DOI:** 10.1186/1755-7682-3-12

**Published:** 2010-06-21

**Authors:** Takashi Matsui, Keiko Ichihara-Tanaka, Chen Lan, Hisako Muramatsu, Toshiharu Kondou, Chizuru Hirose, Sadatoshi Sakuma, Takashi Muramatsu

**Affiliations:** 1 Cell Signals Inc, 3-29-18 Youkoudai, Isogoku, Yokohama 235-0045 Japan; 2 Department of Health Science, Faculty of Psychological and Physical Science, Aichi Gakuin University, 12 Araike, Iwasaki-cho, Nisshin, Aichi 470-0195, Japan; 3 Department of Nutritional Health, Faculty of Psychological and Physical Science, Aichi Gakuin University, 12 Araike, Iwasaki-cho, Nisshin, Aichi 470-0195, Japan; 4 Department of Biochemistry, Nagoya University Graduate School of Medicine, 65 Tsurumai-cho, Showa-ku, Nagoya 466-8550, Japan; 5 Chuden CTI Ltd, Higashisakura 1st Bld., 1-3-10 Higashisakura, Higashi-ku, Nagoya 461-0005, Japan

## Abstract

**Background:**

Midkine is a heparin-binding cytokine and is involved in etiology of various diseases. Thus, midkine inhibitors are expected to be helpful in treatment of many diseases.

**Methods:**

We developed a simple assay for midkine activity based on midkine-dependent migration of osteblastic cells. Midkine inhibitors were searched as materials that inhibit this midkine activity. To develop peptides that inhibit midkine activity, we constructed models in which C-terminal half of midkine interacted with α_4_β_1_-integrin. Low molecular weight compounds which are expected to bind to midkine with high affinity were searched by *in silico *screening with the aid of Presto-X2 program.

**Results:**

Among peptides in putative binding sites of midkine and the integrin, a peptide derived from β_1_-integrin and that derived from the first β sheet of the C-terminal half of midkine significantly inhibited midkine activity. Two low molecular weight compounds found by *in silico *screening exhibited no toxicity to target cells, but inhibited midkine activity. They are trifluoro compounds: one (PubChem 4603792) is 2-(2,6-dimethylpiperidin-1-yl)-4-thiophen-2-yl-6-(trifluoromethy)pyrimidine, and the other has a related structure.

**Conclusions:**

The assay procedure is helpful in screening midkine inhibitors. All reagents described here might become mother material to develop clinically effective midkine inhibitors.

## Background

Midkine is a heparin-binding cytokine of molecular weight 13 kDa [1-3]. It enhances growth, survival and migration of various target cells. Midkine has around 50% sequence identity with pleiotrophin, and the two factors exhibit overlapping roles in many cases [1,4]. Midkine is also involved in initiation or progression of many pathological status, such as tumor invasion [5] rheumatoid arthritis [6], experimental autoimmune encephalitis [7], adhesion after surgery [8], neointima formation of the blood vessel [9], hypertension [10], and renal injury after ischemia [11], exposure to chemotherapeutic reagent [12] and diabetes [13]. Antisense oligonucleotides or siRNAs to midkine exhibit therapeutic effects in animal experiments concerning tumor growth [5,14-16], ischemic renal failure [17], neointima formation [18,19], adhesion after surgery [20], and antibody-induced arthritis [20] Polyclonal antibodies to midkine inhibit growth of Wilms' tumor cells *in vitro *[21]. Peptides or low molecular weight compounds are also expected to inhibit midkine activities, and in many cases these inhibitors might be superior to antisense oligoDNA or siRNA because of the ease in the administration methods.

In the present investigation, at first we developed a convenient method to screen midkine inhibitors. Then, applying the assay method, we tried to develop midkine inhibitors, namely peptides and other low molecular weight compounds.

Midkine is composed of two domains held by disulfide bridges. The C-terminal half domain is principally responsible for midkine activity and its heparin binding capability [22]. Two heparin binding sites are present in the C-terminal half [23]. Especially, Arg 81 has been identified to be important in heparin binding and midkine-induced neurite outgrowth and migration of neurons [24,25]. Thus, we searched for peptides and low molecular weight compounds, which bind to the C-terminal half.

A rationale to obtain midkine inhibitory peptide should be based on midkine-receptor interaction. Midkine receptor is a molecular complex containing proteoglycans such as receptor-like protein tyrosine phosphatase ζ[25], low density lipoprotein receptor-related protein [26] and integrin α_4_β_1 _or α_6_β_1 _[27]. A peptide sequence derived from low density lipoprotein receptor-related protein has already been utilized to develop an inhibitor of midkine [28]. In this study we were interested in interaction of midkine with α4β1 integrin.

## Methods

### Materials

Human midkine was produced in yeast as described before [29]. Candidate inhibitory peptides were synthesized by Peptide Institute, Osaka, Japan, as trifluoroacetate salts. Low molecular weight compounds for screening of midkine inhibitors were purchased from ChemDiv (San Diego, CA).

### Assay of midkine activity

Midkine activity was determined by promotion of migration of UMR106 (ATTC No. 1661) cells [30]. Chemotaxicell (pore size 8 micron, Kurabo Industries Ltd., Japan) was coated with 20 μg/ml of midkine at the lower surface of the filter for 1 h, and washed with Dulbecco's phosphate-buffered saline (PBS) twice. UMR106 cells (2 × 10^5 ^cells in 0.2 ml of Dulbecco's modified Eagle's medium with 0.3% bovine serum albumin) were added to the upper chamber. The lower chamber contained 0.5 ml of the same medium. Cells were cultured for 4 h. The inner Chemotaxicell was cleaned with PBS, and cells were fixed with 100% methanol at room temperature for 20 min. The inner cell was cleaned with cotton bar, and the migrated cells were stained with 1% crystal violet at room temperature for 30 min., and were washed with H_2_O. The cells were extracted with 0.2 ml of 1% SDS with 1% Triton X-100 for 1 h upon agitation. Then extract of 0.15 ml was transferred to a 96 well plate and OD 590 nm was determined. Upon inhibition assay using low molecular weight compounds, they were dissolved in ethanol and were added to the lower chamber so that the final ethanol concentration was 2%. The control contained also 2% ethanol in this occasion. Inhibition assay using peptides was performed by adding them dissolved in H_2_O. Assay was done in duplicate.

### Other methods

Binding activity to midkine was evaluated in the BIACORE 3000 System (GE Healthcare, UK Ltd). A CM5 sensor chip coated with carboxymethyldextran was activated by injecting a solution (1:1) of 200 mM N-ethyl-N'-(3-dimethylaminopropyl) carbodiimide hydrochloride and 100 mM N-hydroxysuccinimide at a flow rate of 10 μl/min. A 70 μl aliquot of midkine solution (50 μg/ml in 10 mM acetate buffer, pH 5.0) was injected for immobilization. The remaining activated N-hydroxysuccinimide ester groups were blocked by injecting 1 M ethanolamine hydrochloride/NaOH, pH 8.5, and washed with 10 μl of 1 M NaCl. Peptides (5 μg/ml) in the running buffer (10 mM HEPES-NaOH, pH 7.4, containing 0.15 M NaCl and 0.0005% Tween 20) were injected into the flow cell, and the change in resonance units was recorded. Binding assays were performed at 25°C with a constant flow rate of 10 μl/ml in both association and dissociation phases.

Docking of α_4_β_1_-integrin with midkine was performed by the Greenpepper program (Chuden CTI) and docking of low molecular weight compounds in ZINC library http://zinc.docking.org/ with midkine was performed by the Presto-X2 program (National Institute of Advanced Industrial Science and Technology, Tsukuba, Japan).

## Results

### Design of midkine inhibitory peptides based on interaction of midkine with integrin

The three dimensional structure of α_4_β_1_integrin was deduced by homology modeling employing the structure of α_V_β_3_-integrin [31]. The three dimensional structure of midkine was determined by NMR [23]. The possible three dimensional structures were deduced for the complex of the C-terminal half domain of midkine and α_4_β_1_-integrin. Among 50 higher scores one, in 29 cases interactions occurred in relatively exposed area, namely, either the interacting site in the α-chain is within 450 amino acid residues from the N-terminal and or interacting site in the β-chain is within 250 amino acid residues from the N-terminal. From the amino acid residues expected to be in the contact sites in the 29 cases, and from the homology of α_V_β_3_- and α_4_β_1_-integrins, we searched stretch of amino acids that have multiple contact sites and expected to be in α-helix or β-sheet structure. The majority of contact sites in the integrin were in expected random coil structures, but sequences in Table 1 were in the expected α-helix orβ-sheet structures.

**Table 1:** Binding of integrin-derived peptides to midkine

Name	Sequence	Origin	Expected structure	Binding constant to midkine (μM)
KQ9	KQNQVKFGS	α_4 _247-255	α-helix	0.20
AM7	AMETNLV	α_4 _359-365	β-sheet	0.95
MQ7	MQSTIRE	α_4 _335-341	β-sheet	0.23
TF8	TFSQRIEG	α_4 _419-426	β-sheet	0.14
KA11	KANAKSCGECI	β_1 _29-39	α-helix	0.27
IE15	IENPRGSKDIKKNKN	β_1 _80-94	β-sheet	0.30
PA9	PAKLRNPCT	β_1 _200-208	α-helix	0.45

The expected binding sites in the midkine C-terminal half to α_4 _β_1 _integrin were mostly in the three anti-pararel β-sheets [23]. Thus the following peptides in the β-sheets were selected as candidate peptide inhibitors derived from midkine: YKFENWGACDGG (YK12), GTGTKVRQGTL (GT11), and QETIRVTKPC (QE10).

By surface plasmon assay, all of the integrin-derived peptides were found to have significant binding activity to midkine (Table 1). Then, we tested whether these peptides inhibited midkine activity.

As the result YKFENWGACDGG (YK12) and KANAKSCGECI (KA11) were found to inhibit the migration activity when added at the concentration of 1 mg/ml to the lower chamber (Table 2). KA11 is derived from N-terminal region of β1-integrin (Table 1). Midkine-derived YK12 is in an externally located β-sheet. When the peptides YK12 and KA11 were added to culture of UMR106 cells at the concentration of 1 mg/ml, no toxicity was observed, although inhibition of cell-substratum adhesion was observed in a certain occasion.

**Table 2:** Inhibitory activities of synthetic peptides and low molecular weight compounds to midkine-dependent migration.

Materials	Concentration	Migration activity (%)
None		100

Peptides		
YK12	1 mg/ml	44.0 ± 10.5
GT11	1 mg/ml	156 ± 6
QE10	1 mg/ml	99.5 ± 10.6
KQ9	1 mg/ml	107 ± 15
AM7	1 mg/ml	99.5 ± 6.9
MQ7	1 mg/ml	107 ± 2
TF8	1 mg/ml	105 ± 24
KA11	1 mg/ml	43.7 ± 12.8
IE15	1 mg/ml	113 ± 10.1
PA9	1 mg/ml	69.4 ± 46.1
Low molecular weight compounds		
Compound I	0.146 mg/ml	43.8 ± 10.2
Compound II	0.10 mg/ml	53.6 ± 4.1

### Search of low molecular weight compounds with inhibitory activity

**Figure 1:**
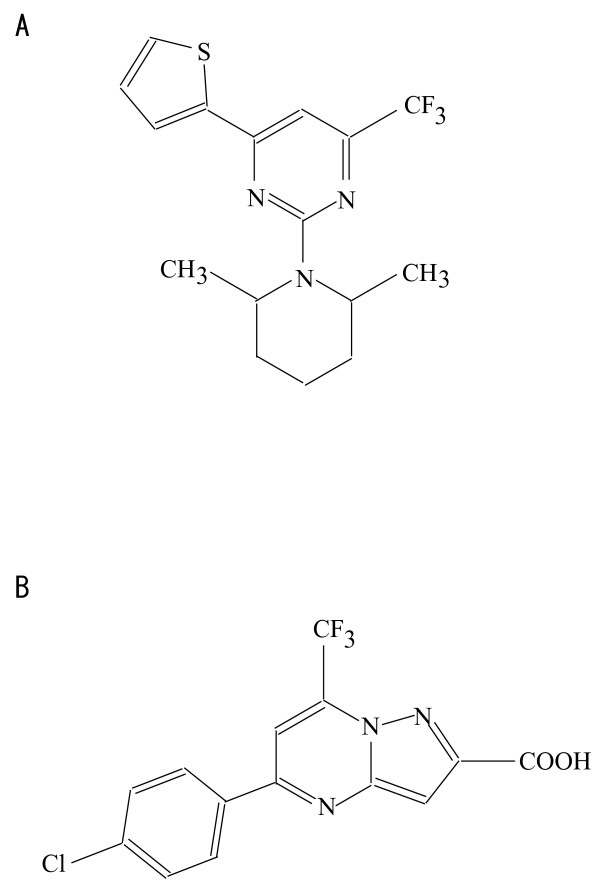
**Structure of compounds with midkine inhibitory activities**. A, Compound I; B, Compound II.

**Figure 2:**
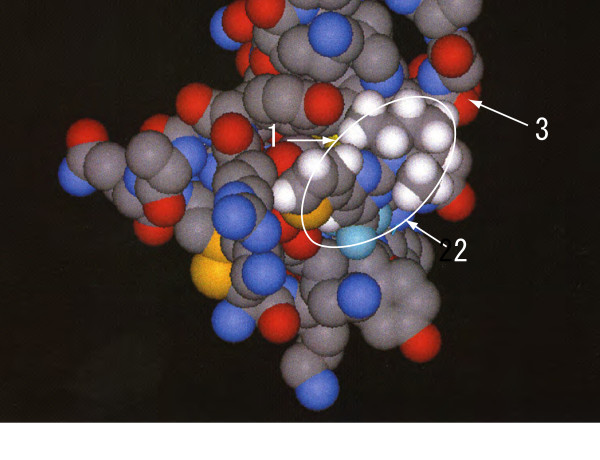
**A model of binding of Compound I to midkine as deduced by Presto X-2 program**. White circle shows Compound I, and 1, 2 and 3 represent Arg81, Lys65 and Phe66, respectively.

The ZINC library of drug-like compounds were surveyed *in silico *for possible binding activity to C-terminal half of midkine. We selected 100 compounds, which were available from ChemDiv, one of the suppliers of drug-like compounds, and were among 531 compounds with the highest binding score in 300,000 compounds so far surveyed. Among the 100 compounds purchased, 10 compounds were soluble in ethanol at 1 mg/ml, and were not toxic to L cells after culturing for 24 h at the concentration of 40 μg/ml. These 10 compounds were surveyed for the activity to inhibit midkine activity. Consequently, two compounds, Compound I [2-(2,6-dimethylpiperidin-1-yl)-4-thiophen-2-yl-6-(trifluoromethy)pyrimidine; PubChem:4603792;ZINC00213280] and Compound II [ZINC00184372; no PubChem number, no official name] (Fig. 1) were found to significantly inhibit the activity (Table 2). They showed no toxicity to the target cells at the concentration used for the assay. The two compounds share a common structure, CF_3 _(Fig. 1). In the model of the interaction of Compound I with the C-terminal half of midkine, CF_3 _faces Lys 65 and Phe 66, which are in the first β-sheet (Fig 2). In the space filling model, compound I, the area of which is shown by a white line, also covered Arg. 81 (marked with an arrow as 1), which forms a key heparin-binding site [23] (Fig. 2). Mutation of Arg 81 leads to loss of neurite-promoting activity of midkine [24]. Compound II is also predicted to bind to midkine in a manner very similar to Compound I, while coverage of Arg 81 was only partial. (data not shown).

## Discussion

A convenient method has been developed to survey materials that inhibit midkine activity. This method has been effective to screen peptides and low molecular weight compounds with the inhibitory activity. An aptamer, which has midkine inhibitory activity and inhibits development of experimental autoimmune encephalitis, was also found using the method [7].

Concerning peptides and low molecular weight compounds with midkine binding and midkine inhibitory activity, these are expected to bind mainly to the first β-sheet of the C-terminal half domain. Probably, this strand is exposed and is easier to be bound by the inhibitors, and through this binding, R81, which is a key amino acid in midkine activity and comprises a heparin binding site [23,24], is expected to be covered by the inhibitor. In addition, a possibility is not excluded that the first β-sheet itself plays a key role in midkine activity, such as in biding to integrins. Indeed a peptide derived from the first β-sheet was found to inhibit midkine activity. It is noted that the first β-sheet has Trp 69, which forms βC1 bulge with Arg81, the key residue in heparin-binding, which is in the centrally located β-sheet [23].

The inhibitory activity of peptides and low molecular weight compounds described here is not potent, and further elaboration is required to obtain materials of potential clinical utility. However, we think that the present material might even become seed materials. As an example, we noted that a methyl group in Compound I, a midkine inhibitory material, is expected to be located near to Arg 81 (marked with an arrow as 1 in Fig. 2), a key amino acid in midkine activity. Thus, conversion of the methyl group to carboxyl group might enhance the affinity of the compound to midkine, and increases the inhibitory activity.

## Conclusions

A simple assay procedure was developed for screening of midkine inhibitors. Inhibitory materials, especially low molecular weight compounds found by the present study are expected to become mother compounds to develop clinically applicable midkine inhibitors.

## Abbreviations

PBS: Dulbecco's phosphate-buffered saline

## Competing interests

The authors declare that they have no competing interests.

## Authors' contributions

TMa designed and performed an assay to screen midkine inhibitors, KI searched for low molecular weight compounds suitable for screening from candidate compounds, CL determined binding activity of synthetic peptides to midkine, HM designed candidate peptides with midkine inhibitory activity, TK designed the system of homology modeling and docking and performed *in silico *screening with CH, CH performed homology modeling, docking and *in silico *screening, SS designed over-all strategy to develop midkine inhibitors with TMu, and played key roles in preparing the second draft of the manuscript, TMu Designed over-all strategy to develop midkine inhibitors with S. S., designed *in silico *screening of low molecular weight compounds with midkine binding activity, and prepared the first draft of the manuscript. All authors contributed to the preparation of the manuscript and approved the final version.
